# Books and Pamphlets Received

**Published:** 1886-11-20

**Authors:** 


### BOOKS AND PAMPHLETS RECEIVED.

*Diseases of the Stomach and Intestines*; a Manual of Clinical Therapeutics for the Student and Practitioner. By Prof. Dugarden Beaumetz, Physician to the Cochin Hospital; Member of the Academy of Medicine and of the Council of Hygiene; Editor-in-chief of the Bulletin General de Therapeutique, etc. Translated from the fourth French edition by E. P. Hurd, M. D., President of the Essex North District Medical Society; Member of the Massachusetts Medical Society; Physician to Anna Jaques Hospital, etc. With illustrations and one chromo-lithograph. New York: William Wood & Co. 1886.

We have discussed "Lavage and gavage of the stomach," "Divisions of Dyspepsias," "Putrid Dyspepsia," "Acid Dyspepsia," "Atonic Dyspepsia," "Treatment of Vomiting," "Treatment of Neuroses of the Stomach," "Intestinal Dyspepsia," "Dyspepsia of Infants," "Ulcer and Cancer of Stomach," "Hygienic treatment of Constipation"—all these, with many other important conditions, are ably and practically considered. We regard this as an exceedingly valuable work. It is neatly printed, and contains 387 octavo pages.

Touching the above work, Dr. Hurd, the translator, remarks that "Few persons of our day have more completely realized in themselves, and in their surroundings, the conditions for successful clinical study and teaching than the author of this work. His hours of labor are divided between the laboratory, the hospital, his large private clientele, his books, and the several learned societies to which he belongs. His early training was under such masters as Behier, Velpeau, Frousseau, Chassaignac, Magendi, and his daily associates are the magnates in the profession of France."

The first five chapters of the book are devoted to regimen, and herein many useful and practical suggestions. The chapters on the various intestinal diseases will be found very interesting and instructive.

*Rheumatism and Gout and some other disorders.* By Morris Longstreth, M. D., one of the Attending Physicians of the Pennsylvania Hospital; Lecturer on Pathological Anatomy at Jefferson Medical College, Philadelphia, Pa. New York: William Wood & Co. 1882.

This is a work of 280 octavo pages, in which the various forms and varieties of rheumatism are treated, under the several heads of Causes, Pathology, Description and Course, Individual Symptoms and Peculiarities, Condition of Skin and its functions, Genito-urinary Apparatus, The Urine, Complications or extra articular localizations, Nervous Complications, Morbid Anatomy, Diagnosis and Prognosis, Treatment, Chronic Articular Rheumatism, Gonorrhœal Rheumatism and Gout. The work contains a pretty full review of the present views and literature on the subject of rheumatic diseases.

*A Text-book of Human Physiology;* including Histology and Microscopical Anatomy, with special reference to the requirements of practical medicine. By Dr. L. Landois, Professor of Physiology and Director of the Physiological Institute, University of Griefswald. Second American edition. Translated from the fifth German edition, with additions, by William Sterling, M.D., Sc. D., Brackenbury Professor; Examiner in the Honours School of Science, University of Oxford. Five hundred and eighty-three illustrations. Philadelphia, Pa.: P. Blakiston, Son & Co. 1886.

The above work contains 922 large octavo pages, and is adapted to both the student and professor. We regard it as among the most able and best of the many valuable works on Physiology which have been published of late years. It claims to form a bridge between Physiology and the Practice of Medicine. After describing the normal processes, the pathological conditions are briefly and ably referred to. The histological features of the work are very instructive and acceptable to the student and especially to the teacher. The work is fully up to the latest advances. The illustrations are excellent, and the work is in all respects eminently able, instructive and practical.

*Diseases of Spinal Cord.* By Byrom Bramwell, M. D., F. R. C. S. (Edinburgh), Lecturer of the Principles and Practice of Medicine and on Medical Diagnosis in the Extra Occasional School of Medicine, Edinburgh; Pathologist to the Edinburgh Royal Infirmy; Additional Examiner in Clinical Medicine in the University of Edinburgh; late Physician and Pathologist to the Newcastle-on-Tyne Infirmary, etc. Fifty-three colored plates and 102 fine wood engravings. Second edition. New York: William Wood & Co. 1886.

The above is an able and carefully prepared work on spinal affections, a class of diseases which are confessedly obscure, and about which the profession are, as a rule, but poorly informed. This work should find its way into every Medical library and be carefully studied by the practitioners throughout the land. It contains 298 octavo pages, neatly printed.

The *Century Magazine* comes regularly among our exchanges. It is now regarded as standing at the very head of literary periodicals, not only in America but in the World.

